# Successful treatment of palmoplantar pustulosis with topical ruxolitinib

**DOI:** 10.1111/ddg.15854

**Published:** 2025-08-23

**Authors:** Neda Cramer, Phoebe Wellmann, Michael P. Schön, Rotraut Mössner

**Affiliations:** ^1^ Department of Dermatology Venereology and Allergology University Medical Center Göttingen, Germany

**Keywords:** JAK inhibitors, palmoplantar pustulosis, psoriasis

Dear Editors,

Palmoplantar pustulosis (PPP) is a chronic inflammatory skin disease which is considered a variant of pustular psoriasis and is characterized by recurrent sterile pustules, erythema, and scaling on the palms and soles.^1^ Due to its chronic and relapsing nature and the associated pain, it can cause functional disabilities and impair the patients’ quality of life.^2^ A differential diagnosis to PPP is psoriasis cum pustulatione, where pustules arise within or at the edge of psoriasis plaques.^1^ Treatment of PPP is challenging. To date, no therapeutic standards or published guidelines are available. Treatment options include topical corticosteroids, systemic retinoids, phototherapy, and other systemic therapies approved for plaque psoriasis or psoriatic arthritis, such as methotrexate, cyclosporine, biologics, and Janus kinase (JAK) inhibitors.^3^, ^4^ There is increasing evidence from case reports and case series suggesting an excellent efficacy of oral JAKi (JAK inhibitors) in PPP,^5^, ^6^, ^7^ while, to our knowledge, no reports on topical therapy with JAKi have been published.

We report on a patient with PPP who was refractory to topical and UV therapy and responded successfully to off‐label treatment with topical ruxolitinib, a selective JAK 1/2 inhibitor.

A 66‐year‐old female presented with a 10‐month history of pruritic palmoplantar skin lesions characterized by erythema, scaling, and pustules, with pustules also occurring in areas without psoriatic plaques (Figure 1a). A diagnosis of PPP was made. Topical corticosteroids, including mometasone furoate and betamethasone valerate under occlusion, had been used since disease onset but showed insufficient therapeutic effect, as did phototherapy with psoralen plus ultraviolet A (PUVA). The patient was a smoker (20 pack‐years). Family history for skin diseases was unremarkable. She also had mild seronegative rheumatoid arthritis affecting the small finger and toe joints, with normal C‐reactive protein levels, and was treated with ibuprofen as needed. Following the onset of psoriatic disease, joint symptoms occurred alongside the skin manifestations and were subsequently interpreted as psoriatic arthritis.

**FIGURE 1:**
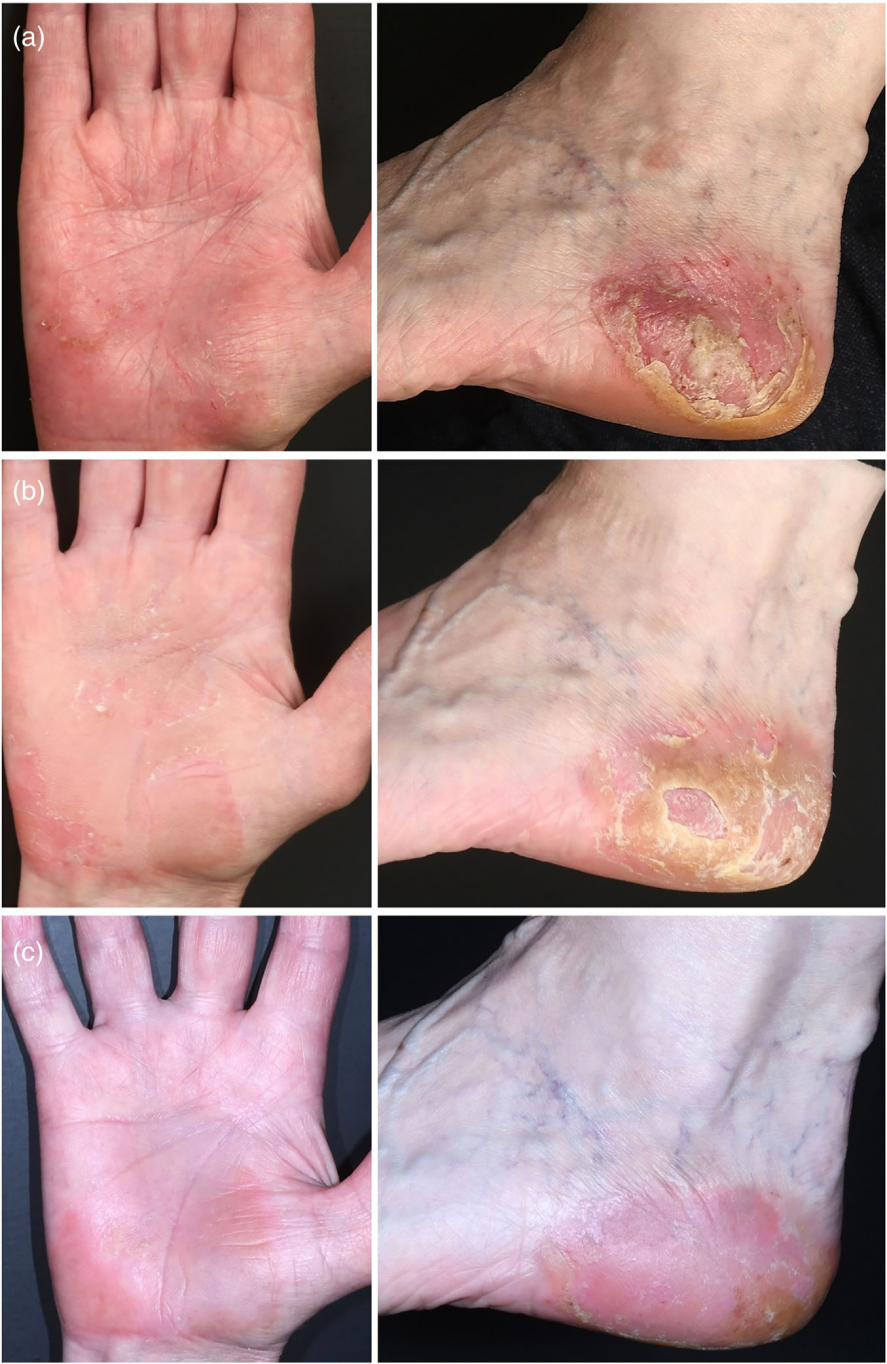
Clinical images of (a) the right palm and left foot before therapy, (b) after 8 weeks, and (c) after 10 weeks of treatment with topical ruxolitinib.

On admission, the *Palmoplantar Pustulosis Area and Severity Index* (PPPASI) was 6.2 out of 72 (Figure 1a), pain and itching were reported as 9/10 on the numerical rating scale (NRS) and the *Dermatology Life Quality Index* (DLQI) was 17 out of 30, indicating a high impact on quality of life. Due to the poor response to conventional topical therapies and the patient's refusal of systemic therapies, she was treated off‐label with ruxolitinib cream (Opzelura™, 15 mg/g twice daily, applied as a thin layer).

After 3 weeks of therapy, the skin lesions had improved and the PPPASI had decreased to 3.2. The itching, pain, and quality of life had also improved (NRS 5/10 and 3/10, respectively; DLQI 12). To enhance the therapeutic effect, the medication was applied twice daily for 1–2 hours under an occlusive dressing made of cling film. After 8 weeks, the PPPASI decreased to 2.0 (Figure 1b). Itching, pain, and quality of life also improved (NRS 5/10 and 1/10, respectively; DLQI 7). Due to increased skin softness and tenderness under twice‐daily occlusive treatment, the frequency of occlusive application was reduced to once daily for 1–2 hours. After 10 weeks, the PPPASI was 0.8 (Figure 1c), while itching and pain were absent (NRS 0/10 for both parameters). No systemic adverse events occurred. However, the patient felt somewhat restricted by the cumbersome application and the time required for the cream to be absorbed.

Topical JAKi are an emerging class of drugs for the treatment of inflammatory skin diseases. Approved topical JAKi in the EU are the pan‐JAKi delgocitinib for moderate to severe chronic hand eczema in adults,^8^ and ruxolitinib for non‐segmental vitiligo with facial involvement in patients aged ≥ 12 years.^9^ Data from clinical studies on topical ruxolitinib indicate efficacy in atopic dermatitis, vitiligo, psoriasis, and lichen planus.^10^ It is approved by the FDA (U. S. Food and Drug Administration) for the treatment of mild to moderate atopic dermatitis in patients aged ≥ 12 years.^11^ A pharmacokinetic study in minipigs showed that topically applied ruxolitinib was only minimally absorbed systemically and that its concentration in the skin was higher compared to systemic application.^12^

Thus, topical JAKi appear to be a promising therapeutic option for inflammatory skin diseases such as PPP and further clinical studies are warranted to evaluate its potential benefit in this indication.

## CONFLICT OF INTEREST STATEMENT

R.M. has been an advisor and/or received speakers’ honoraria and/or received grants and/or participated in clinical trials of the following companies: AbbVie, Amgen, Almirall, Biogen IDEC, Boehringer‐Ingelheim, Celgene, Janssen‐Cilag, Leo, Lilly, Moonlake, MSD SHARP & DOHME, Novartis, Pfizer, UCB. M.P.S. has been an advisor and/or received speakers’ honoraria and/or received grants and/or participated in clinical trials of the following companies: AbbVie, Almirall, Biogen, Boehringer‐Ingelheim, BMS, Celltrion, Janssen‐Cilag, Leo, Lilly, Novartis, Scinai, UCB.

P.W. and N.C. report no conflicts of interest.
